# Pesticide Availability and Usage by Farmers in the Northern Region of Ghana

**DOI:** 10.5696/2156-9614-9.23.190906

**Published:** 2019-08-06

**Authors:** Ziblim A. Imoro, Joshua Larbi, Abudu B. Duwiejuah

**Affiliations:** 1 Biodiversity Conservation and Management, Faculty of Natural Resources and Environment, University for Development Studies, Nyankpala Campus, Tamale, Ghana; 2 Ecotourism and Environmental Management, Faculty of Natural Resources and Environment, University for Development Studies, Nyankpala Campus, Tamale, Ghana

**Keywords:** crop farmers, health, herbicide, insecticide, pesticide, pollution

## Abstract

**Background.:**

Over and improper use of chemical pesticides can have adverse effects on the environment, human health, and social capital.

**Objectives.:**

The present study investigated pesticides available in the market, as well as handling and usage of pesticides by farmers in the Northern Region of Ghana.

**Methods.:**

Cluster sampling was used to select 20 communities from the Tolon District. Simple random sampling was then used to select 5 households from each community and one farmer from each household, giving a sample size of 100 farmers.

**Results.:**

The survey identified 39 agrochemical shops in the Tamale Metropolis. Thirty-six different pesticides were identified on the market, mainly comprised of insecticides and herbicides. The predominant active ingredients were cypermethrin and glyphosate in insecticides and herbicides, respectively. The survey revealed 18 kinds of pesticides commonly used by the farmers on their fields, with atrazine being the most commonly used herbicide (42%) and Lambda Super 2.5 EC the most commonly used insecticide (50%). The study also revealed that 64% of the respondents disposed of their empty pesticide containers indiscriminately and 44% stored used and unused pesticides in their bedrooms.

**Conclusions.:**

The mode of disposal of used pesticides containers and storage of pesticides by the respondents contribute to human health and environmental hazards. Measures to educate farmers on pesticide usage and storage to help accomplish the target of environmentally friendly and sustainable agricultural production should be taken by the appropriate authorities.

**Participant Consent.:**

Obtained

**Competing Interests.:**

The authors declare no competing financial interests.

## Introduction

Pesticides have been used for crop protection since at least 2000 BC. Elemental sulfur dusting was the first known pesticide used in ancient Sumer approximately 4,500 years ago in prehistoric Mesopotamia.^1^ Pesticides are substances of a chemical nature used to control organisms that are harmful. Their use in agriculture can adversely affect the environment, ecosystems and human health.^2^ Pesticides are sub classified by the organism type they are intended to control such as biocides, insecticides, fungicides, herbicides, rodenticides, pediculocides, molluscicides, nematicides, and plant growth hormones.^3^,^4^

Modern agriculture is largely dependent on effective chemicals for crop protection, improving crop yield, and cost-effective production. In Ghana, pesticide use by farmers for the control of pests and weeds and harvested crops preservation has increased in recent years.^5^However, the use of pesticides can have harmful effects on consumers, farmers, and traders involved in the food supply chain.^6^ Indiscriminate use of pesticides in the past has contaminated the environment through accumulation in air, feed, food, soil, and water bodies.^7^ In Ghana, farmers' poor knowledge of pesticide types, their use and attendant risks, ineffective enforcement of pesticide regulations by the government and robust incentives amongst pesticide users and traders to create income has led to an increased use of adulterated, mislabelled and cheap pesticides.^8^

Many reports detail the effects of pesticides on the health of farmers and the environment.^9^,^10^ Most studies on leukemia and non-Hodgkin lymphoma have shown a positive relationship with pesticide exposure and conclude that heavy use of pesticides should be decreased.^11^

Other adverse effects associated with pesticide exposure include birth defects, fetal death, neurological and neuro-developmental disorders.^12^,^13^ There is also growing concern about the improper disposal of pesticide waste as it can create serious threats to humans and the environment.^14^

In the Northern Region of Ghana, pesticides are used by most farmers to boost and increase production of food and crop yields. However, over use and improper use of chemical pesticides can have adverse effects on the environment, human health, and social capital. In addition, most farmers have no formal education and do not follow precautions for pesticide application and usage, increasing adverse risks to the environment and humans. This study aimed to identify the various pesticides available on the market, determine active ingredients, identify the types mostly used by farmers, assess storage of used and unused pesticide by farmers and assess mode of disposal of empty pesticide containers in the study area.

## Methods

The survey was conducted in the Tamale Metropolis where most farmers buy their agro-chemicals and the Tolon District, which is one of the farming districts in the region. Tamale Metropolis lies between longitude 0°46′ and 0°59′ W and latitude 9°15′ N within the savannah woodland region. Tamale has a population of 371,351 and is the capital of the Northern Region, Ghana.^15^ Tolon District is located at longitude 20°50′ W and latitude 10°20′ N with Tolon as its capital. It lies within the Guinea savannah agro-ecological zone with a typical formation of fire-resistant trees and shrub-grassland vegetation. Tolon has a population of 135,084.^15^ The inhabitants of this area are primarily mixed farmers growing crops and raising livestock. The most commonly cultivated crops are cassava, cowpea, groundnut, maize, kenaf, okro, rice, tomatoes, sorghum, pepper and yam. The rainfall pattern is unimodal with annual average of 900–1100 mm and a mean annual temperature of 25.7°C. Geologically, the soils are mainly comprised of Voltaian formation, characterized by shallow depth and a cemented layer of iron pan. The soils type ranges from sandy-sandy loam with a typical low cation exchange capacity.

### Sampling

Data were collected from two categories of respondents; 1) agro-chemical shop attendants or distributors, and 2) farmers. The research was conducted from October 2010 to March 2011. Purposive sampling was used to identify shops stocking agro-chemicals in the Tamale Metropolis. Thirty-nine shop attendants and distributors were interviewed using a semi-structured questionnaire developed by the authors. The questionnaire can be found in Supplemental Material. The purpose and confidentiality of the study was explained to each subject before each interview.

Cluster sampling was used to select 20 communities from the Tolon District. Simple random sampling was then used to select five households from each community and one farmer from each household, giving a sample size of 100 farmers. All farmers selected for the study agreed to participate. The selection of 100 farmers was based on their engagement in farming activities and usage of pesticides. Similar responses were being repeated during the data collection, indicating saturation. This sampling was considered to be adequate to cover the full range of pesticide handling and application practices by farmers in the Tolon District. A semi-structured questionnaire developed by the authors was then administered to the selected farmers. The questionnaire can be found in Supplemental Material. Personal observation was employed to observe chemicals available in the shops, pesticides used by famers and mode of disposal of pesticides waste containers. Photographs of pesticides that were available at the time of identification were taken with a camera for easy identification. The collected data were processed using the Statistical Package for the Social Sciences (SPSS) version 16. Descriptive statistics such as percentages and frequencies were used for categorical variables and the results are presented in Figures and Tables.

## Results

The survey identified 39 agrochemical shops in the Tamale Metropolis. In the present study, 56.4% of attendants and distributors of these agrochemicals were female and 43.6% were male *(Table 1).* A total of 74.4% were high school graduates, 25.6% had a middle school education, and no respondents had a tertiary level education. Of the farmers interviewed, 40% were between 31–40 years of age, 35% were between 21–30 years of age, and 25% were between 41–60 years of age. In addition, 67% of farmers had no formal education *(Table 1).*

**Table 1:** Demographic Characteristics of Study Participants

	**Number of respondents**	**Percentage (%)**
**Demographic characteristics of shop attendants and distributors**		

Gender of shop attendants and distributors		
Female	22	56.4%
Male	17	43.6%
Level of education		
Senior high school	29	74.4%
Middle school	10	25.6%
**Demographic characteristics of farmers**		
Age of farmers		
21 to 30 years	35	35%
31 to 40 years	40	40%
41 to 60 years	25	25%
Farmers' level of education		
No formal education	67	67%
Formal education	33	33%

### Pesticides available on the market

The study found a total of 36 different pesticides sold in the metropolis. Herbicides were the most commonly sold and used in the metropolis (58.3%), followed by insecticides (41.7%). The majority (62%) of herbicides were selective (herbicide that targets specific species), and 38% were broad spectrum (herbicide that targets entire groups or species). Glyphosate was the active ingredient found in all of the broad spectrum herbicides, and atrazine, 2, 4, D amide salt and butachlor were the active ingredients in most of the selective herbicides. The study revealed that 26% of these insecticides had cypremethrin as the active ingredient, 20% had lambda-cyhalothrin and 13% had chlorpyrifos. Other active ingredients in the insecticides were lambda, amide salt, carbon/furida, termidor, glyphaside and lambd-acypermethin. The study revealed that most of the herbicides were classified as category III chemicals and all the synthetic insecticides were classified as category II chemicals *(Table 2).*

**Table 2:** Herbicides and Insecticides Identified in Tamale Metropolis

**Active ingredient**	**Brand name**	**Herbicides**	WHO^*^ classification

Glyphosate	Cerites, D-lion, Glygot, Touchdown, Vinash, Sarosates, Nwuranwura and Sunphosate	Broad spectrum	III
Butachlor	Butachcor and Ceres	Selective	III
Atrazine	Aligata, Atraherb 50, Ultrachlore, Sun-Atrazin and Utrazin		III
Pendimethalin	Agristomp		II
Propanil	Propucal plus		II
Isobutylate	Ervextra		III
2, 4-D Amide salt	Amine, Sun, 2, 4 -D and Orizo plus		II

**Active Ingredients**	**Brand Name**	**Insecticides**	

Lambda	Lambda		II
Cypermethrin	Cyperdem, Summitex, Sunhalortharin and Polytrine		II
Lambda-cyhalothrin	Kadmaneb, Kilsect and Kombat		II
Chlorpyrifos	Duraban and Termicost		II
Carbon/furida	Carboden		-
Amine salt	Herberstra		II
Lambda-cypermethrin	Controller		II
Termidor	Hercules		-
Glyphaside	Chemaprid		-

Note: III = slightly hazardous (harmful), II = moderately hazardous (toxic).^16^

^*^ Adapted from The WHO recommended classification of pesticides by hazard and guidelines to classification: 2009.

### Most commonly used pesticides

The study revealed that broad spectrum herbicides with glyphosate as the active ingredient were the most common type used by farmers, while selective herbicides with atrazine as the active ingredient were the farmers' most preferred herbicides. Lambda Super 2.5 EC was the most commonly used (50%) insecticide *(Table 3).*

**Table 3:** Most Commonly Used Herbicides and Insecticides in Tamale Metropolis

**Type of herbicide**	**Active ingredient**	**Number of respondents**	**Percentage (%)**

Condemn	Glyphosate	6	6%
Sarosate	Glyphosate	15	15%
Calliherbe	2,4,D amide salt	8	8%
Atrazine	Atrazine	42	42%
Round-up	Glyphosate	12	12%
Propanele	Glyphosate	5	5%
Stump	2,4,D amide salt	2	2%
K-optimol	2,4,D amide salt	1	1%
Odeneho	2,4,D amide salt	1	1%
Butachlor	Butachlor	1	1%
Ultrachlor	Butachlor	1	1%
Lasso	2,4,D amide salt	1	1%
Gramozine	Glyphosate	2	2%
Power	Glyphosate	2	2%
Alligator 400 EC	2,4,D amide salt	1	1%
Total		100	100%

**Type of insecticide**	**Active ingredient**	**Number of respondents**	**Percentage (%)**

DDT	-	1	1%
Kombata 2.5 EC	Cyhalothrin	32	32%
Karate	Cyhalothrin	17	17%
Lambda Supper 2.5 EC	Cyhalothrin	50	50%

Abbreviation: DDT, dichlorodiphenyltrichloroethane.

### Farmers' access to information on the use of pesticides

**Figure 1:**
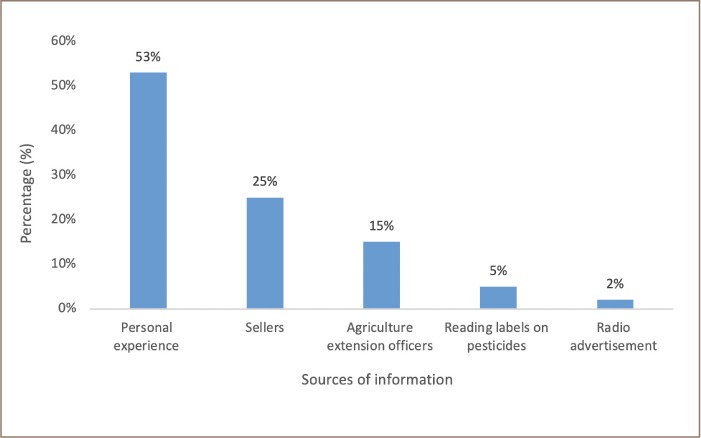
Farmers' access to information on the usage of pesticides

Access to information on the usage of these pesticides by farmers was through personal experience (53%), sellers (25%), agriculture extension officers (15%), reading labels on pesticides (5%) and radio advertisement (2%) *(Figure 1).*

### Pesticide storage

**Figure 2:**
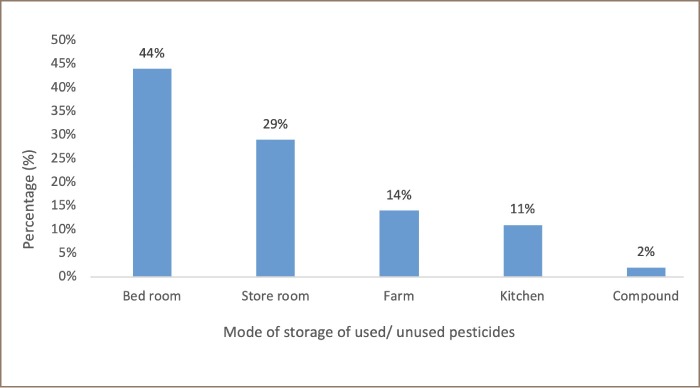
Mode of storage of used / unused pesticides

The present study also questioned farmers about their pesticide storage practices and the majority (44%) reported storing pesticides in their bedrooms. Other farmers (29%) reported that they stored pesticides in storerooms, as well as the kitchen, farms and compounds (*Figure 2*).

### Disposal of empty pesticides containers

**Figure 3:**
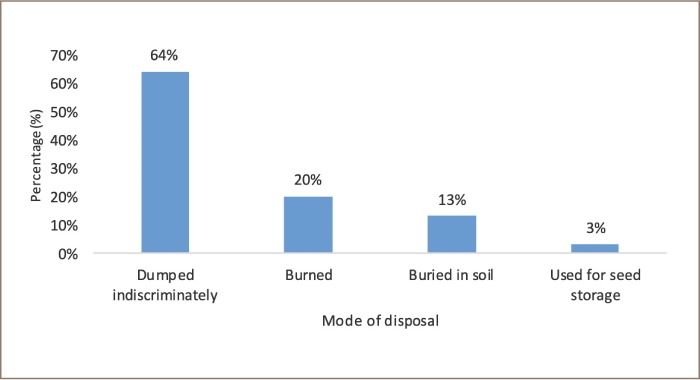
Disposal of empty pesticides containers by farmers

**Figure 4:**
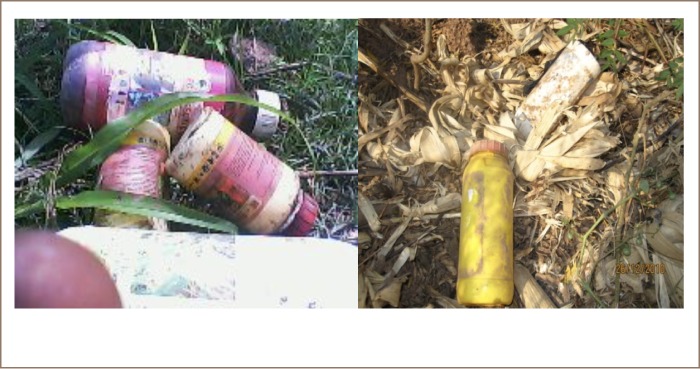
Empty pesticide containers thrown away at the Bontanga irrigation site and Nyankpala community

The study observed that 64% of respondents *(Figure 3)* in the Tolon District discarded pesticides containers indiscriminately *(Figure 4)*; 20% through burning, 13% by burying in soil, and 3% of farmers stored seeds in the empty containers *(Figure 3)*. None of the farmers reported using the empty pesticide containers for storing water. Substantial amounts of pesticides could be left in pesticide containers that are not appropriately disposed of.

### Protective clothing used by farmers and timing of application

**Figure 5:**
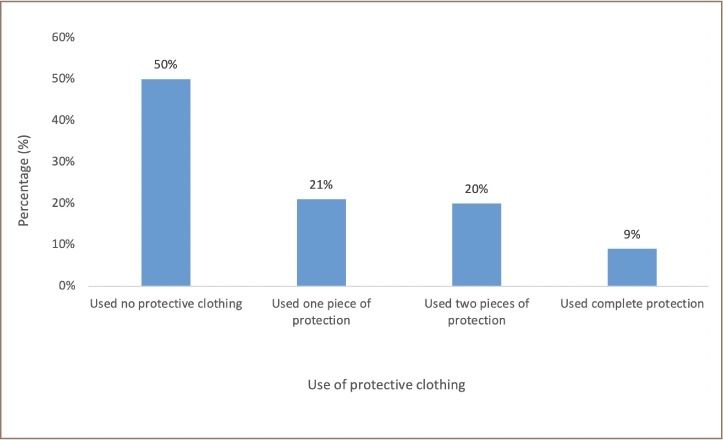
Use of protective clothing by farmers

The present survey found that 50% of farmers used no protective clothing, 9% used a complete set of protective clothing (rubber boots, overalls and mask), 21% used only one piece of protection, and 20% used two pieces of protection when applying pesticides *(Figure 5).* The study showed that 87% of farmers applied pesticides after rain when the soil is moist, which is the encouraged technique, and 13% applied pesticides to dry soil, which is not encouraged. The study observed that 50% of farmers were at risk of pesticide exposure through skin contact, inhalation and ingestion during preparation and application to crops due to failure to use protective clothing.

## Discussion

The low educational level of attendants and distributors in these shops could mean that farmers may not receive appropriate information on the rate, time and method of pesticide application. Misinformation on pesticide application and usage threatens human safety, natural resources, and the environment. Farmers prefer to rely on extension officers, pesticide sellers, and other farmers rather than reading flyers or instructions on the chemicals.^17^ As a result, equipping pesticide sellers with adequate and relevant information through appropriate education on pesticide usage is of utmost importance.^18^

The pesticides most commonly sold in the metropolis were insecticides and herbicides. This is because weed control is the greatest challenge to farmers in Ghana. The majority of the herbicides were selective. Glyphosate was the active ingredient found in all of the broad-spectrum herbicides, and atrazine, 2, 4, D amide salt and butachlor were the active ingredients in most of the selective herbicides.

The active ingredients of the pesticides fell under three categories based on the Pesticide Action Network International classification with regard to long-term effects (atrazine, glyphosate, lambda-cyhalothrin and pendimethalin), environmental toxicity (butachlor, chlorpyrifos, cypermethrin, cyhalothrin, lambda-cyhalothrin and pendimethalin) and acute toxicity (lambda-cyhalothrin).^19^ Insecticides with the active ingredient of cypermethrin were most commonly used by farmers due to their ability to destroy caterpillars and butterflies. Cypremethrin is known to be active against lepidopterous larvae.^20^

The majority of the farmers in the present study had no formal education. Due to this high illiteracy rate, most farmers are not able to follow recommended procedures for pesticide usage, leading to inappropriate usage and resulting environmental and health hazards. Most farmers rely on recommendations from colleagues, their own intuition, and recommendations from extension officers and chemical dealers on modes of application of pesticides. This illustrates that there is a knowledge gap between agriculture extension agents and farmers in the study area. Farmers also depend on pesticide sellers, the majority of which have a high school education and who may not be educated on the appropriate usage of pesticides. This may result in inappropriate handling and usage of pesticides, which can cause serious health and environmental problems.

Broad spectrum herbicides with the active ingredient glyphosate were most commonly used by farmers, along with selective herbicides with atrazine. The insecticide DDT (dichlorodiphenyltrichloroethane), which was banned for agricultural use worldwide under the Stockholm Convention, was reportedly used by some farmers, but was not found on the market. This implies that farmers obtain their pesticides through other channels apart from registered agrochemical dealers in the region.

Farmers reported that they store these pesticides in their bedrooms and kitchen in order to prevent them from being accidentally consumed by children or animals and for theft prevention. Chemicals can volatilize and travel through the air, and storing pesticides in the home makes residents vulnerable to poisoning through inhalation and contaminated food. One of the principal routes that chemicals enter the body is by inhalation.^21^These pesticides have serious effects on human health, as chlorpyrifos is known to be highly toxic to mammals and inhibits the action of certain enzymes.^20^

The study observed that the farmers do not use empty pesticide containers for storing water, suggesting that farmers understand the poisonous nature of these pesticides. According to Ozkan and Heimlich, concentrated pesticide residues that leak from un-rinsed, discarded containers can cause significant environmental contamination, as about three ounces of pesticides is left inside a five-gallon container after normal use.^22^ Improper pesticide waste disposal is a growing concern as it threatens the environment and human health.^14^ Pesticides left in empty containers can be washed into dams, rivers, streams and wells through the action of runoff.^23^Most dams and wells in the study area can be contaminated due to improper disposal of pesticides containers by pesticide users.

Most farmers in the present study were at risk of skin contact, inhalation and ingestion of pesticides during preparation and application to crops, as many did not use protective clothing. Low rates of use of protective clothes by farmers has been reported in other parts of Ghana and beyond.^9^,^10^ Most farmers do not use the safety gloves, masks or other protective gear when spraying these chemicals, resulting in the accumulation of pesticide residues in the bloodstream through dermal and inhalation exposure, which can adversely affect their skin, eyes and respiratory system.^24^ Some farmers reported using partial protective measures, as one study reported that 26% of farmers covered most of their body except their eyes during pesticide application.^25^ Application of category I and II pesticides require farmers to wear appropriate protective equipment that covers the whole body.^26^ The present study found that some farmers are at risk of poisoning as they do not use protective clothing during pesticide application. A study on risk assessment and pesticide usage among workers in the United Kingdom showed that the adoption of safety protection measures is greatly influenced by the socioeconomic status of workers and can be enhanced through appropriate education.^27^

## Conclusions

Insecticides and herbicides were widely available on the market for sale to farmers in the present study. Broad spectrum herbicides with glyphosate and selective herbicides with atrazine as the active ingredient were most commonly used by farmers. Most farmers did not use protective clothing during pesticide application. The farmers practiced indiscriminate disposal of empty pesticide containers. Most of the farmers stored pesticides in their bedroom. Farmers are not adequately informed on human health and environmental hazards that may result from the improper pesticide usage. We recognize that a limitation of the study is that all farmers interviewed were male. It is recommended that the agriculture extension agency, environmental protection agency and other environmental stakeholders organize collaborative educational programs for farmers on the proper use, effective handling, and dangers associated with inappropriate usage of pesticides.
