# Supplementary material for: Pesticide Availability and Usage by Farmers in the Northern Region of Ghana
Source: J Health Pollut. 2019 Aug 6;9(23):190906. doi: 10.5696/2156-9614-9.23.190906 (PMC6711326; doi:10.5696/2156-9614-9.23.190906)
Supplement: Supplementary file 1 [file Imoro_Supplemental_Material_FINAL.docx]

# Supplemental Material

# Questionnaire on Pesticide Availability

I would like to have your views on ‘**Pesticide Availability’** and would be grateful if you could cooperate with me to complete this questionnaire. Any information provided will be kept confidential and used only for research purposes. Thank you.

**Demographic characteristics of shop attendants and distributors**

1. Name of shop ………………
2. Date……………………
3. Name of respondent …………………….
4. Sex: 1. Male [ ] 2. Female [ ].
5. Level of education 1. Informal [ ] 2. Primary/JHS [ ] 3. Middle school [ ] 4. SHS [ ] 5. Tertiary [ ].

**Pesticides available on the market**

1. Types of pesticides sold in the shop 1. Herbicides [ ] 2. Insecticides [ ].
2. Number of different types of herbicide available in the shop………………………..
3. Category of herbicide available in the shop 1. Broad spectrum [ ] 2. Selective [ ].
4. Number of different types of insecticides available in the shop ………………………
5. Category of insecticides available in the shop 1. Broad spectrum [ ] 2. Selective [ ].

# QUESTIONNAIRE

# I would like to have your views on ‘Pesticide Availability and Usage by Farmers in the Northern Region of Ghana’ and would be grateful if you could cooperate with me to complete this questionnaire. Any information provided will be kept confidential and used only for research purposes. Thank you.

**Demographic characteristics of farmers**

1. Community ………………
2. Date……………………
3. Name of respondent …………………….
4. Age range 1. Less than 20 years [ ] 2. 21-30 years [ ] 3. 31-40 years [ ]. 4. 41-60 years [ ]. 5. 61 years and above [ ].
5. Sex 1. Male [ ] 2. Female [ ].
6. Occupation………………
7. Numbers of years of farming experience ……………………………………………
8. Level of education 1. Informal [ ] 2. Primary/JHS [ ] 3. Middle school [ ] 4. SHS [ ] 5. Tertiary [ ].
9. Level of education 1. Informal education [ ] 2. Formal education [ ].

**Pesticides available on the market**

1. How do you get pesticides for use? 1. Personal purchase [ ] 2. By order [ ].
2. Where do you purchase the pesticides? 1. Market [ ] 2. Distributors [ ] 3. Importers [ ] 4. Other ……………………………….
3. Types of pesticides sold in the Metropolis 1. Herbicides [ ] 2. Insecticides [ ].
4. What type of herbicide do you use? ……………………………………………………
5. Category of herbicide 1. Broad spectrum [ ] 2. Selective [ ].
6. What type of insecticides do you use? ………………………………………………
7. Category of insecticides 1. Broad spectrum [ ] 2. Selective [ ].
8. Do you have access to information on the use of pesticides? 1. Yes [ ] 2. No [ ].
9. If yes, how do you obtain access to information on the use of pesticides? 1. Personal experience [ ] 2. Sellers [ ] 3. Agriculture extension officers [ ] 4. Reading labels [ ] 5. Radio advertisement [ ].
10. Where do you store the used or unused pesticides? 1. Bedrooms [ ] 2. Kitchen [ ] 3. Storeroom [ ] 4. Farms [ ] 5. Compound [ ].
11. How do you dispose of empty pesticides containers? 1. Dumped indiscriminately [ ] 2. Burned [ ] 3. Buried in soil [ ] 4. Used for seed storage [ ].
12. Do you use protective clothing during application of pesticides? 1. Yes [ ] 2. No [ ].
13. Use of protective clothing (rubber boots, overalls and mask) 1. No protective clothing [ ] 2. Complete set of protective clothing [ ] 3. Used one piece of protection [ ] 4. Used two pieces of protection [ ].
14. Time of pesticide application 1. After rain [ ] 2. When the soil is dry [ ].
